# A method for failures grouping and priority ranking case study: Operating gas compression plant

**DOI:** 10.1016/j.mex.2021.101268

**Published:** 2021-02-11

**Authors:** Mohamed Hussein M. Faris, Elamin Elhussein, Hassan Osman Ali

**Affiliations:** a Mechanical Engineering Department, Sudan University of Science and Technology, Khartoum 11111, Sudan; b Mechanical Engineering Department, Karary University, Khartoum 11111, Sudan

**Keywords:** Total down time importance, Gas compression plants, Modern maintenance, Maintenance engineering and management, Risk priority number

## Abstract

The gas compression plant is a core and major unit in oil and gas industries that have high gas oil ratio or considerable gas production. Compressed gas is needed as fuel, support processing handling, increase reservoir builds up pressure by gas injection as well as a useful product. Gas plants are critical and dangerous working location and it is classified as a critical zone due to circumstance parameters like high pressure, high temperature, gas specifications and the potential to impact to human health, safety, environment and possibility to impact invested revenues in case of incidents. Therefore, all recorded compression plant operational failures shall be assessed and reviewed in order to decrease the unit down time and increase plant safety and efficiency.

In general, limited studies were conducted in gas plant maintenance management. This paper studied a working gas compression unit in an operating oil and gas field in order to present the followings:

• A model of failures raking and sorting in gas compression plants based on total down time importance (TDTI) grouping.

• A model of failures ranking by using the risk priority number (RPN).

RPN is giving priorities based on associated risk and TDTI as a new method is providing rankings based on maximum contribution to the total occurred down time. Therefore, the study is elaborating to demonstrate these two methods and highlighted the areas of difference which need attention of the owner and the site working team.

## Graphical abstract

**Figure:**
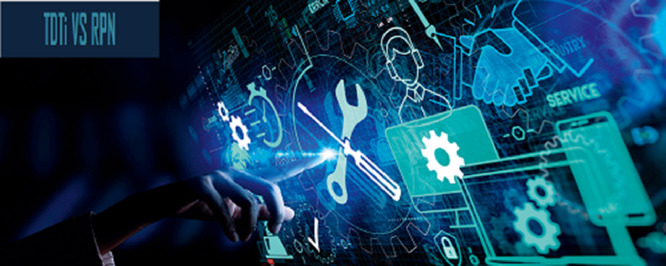

Specifications table

**Table:** 

Subject Area:	Engineering
More specific subject area:	Risk assessment and decision making, Industrial Engineering
Method name:	Total Down Time Importance (TDTI)
Name and reference of original method:	RPN
Resource availability:	Excel Spreadsheet

## Introduction

Investment in gas industries is attractive due to continuous demand of gas and its products as well it has almost a stable global prices. Thus, it is interesting area for studies and researches.

In General, the gas product either received from downhole reservoirs or released from oil treatment process plants. Based on the received amount, the plants will be designed and will depend on the gas specification, amounts & needs. Therefore, based on the required final products and commercial investment, the plant will be designed like refineries, fuel/power generations, flaring and heating systems, raw, etc. Compression unit requires for gas handling to subsequent stages and for gas transportation by using suitable type of compression systems. Gas compression Plant is considered as high critical equipment, because in case of any failure it may has the potential to impact safety, health and environment beside the impact of capital and production loss.

The study considered a new Gas compression plant recently commissioned in 2016 but after four operating years, it showed high records of downtime (34 recorded un-planned failures) compared to the designed duty time and unit quality (current running hours is 20,000). Nevertheless, the working team are following the manufacturer and common maintenance practice. Therefore, it is required to rank the list of failure based on the priorities as baseline.

These priorities can be based on risk or based on the failures which have more contribution to overall down time. Thus, this study demonstrates the both methods of ranking by total down time importance (TDTI) and also list using risk priority number (RPN) determination to identify the most failures need more focusing and high attention to resolve to increase compression plant efficiency by decreasing the causes of the down time.

## Literature review

Recently, some studies conducted for compressions plants to increase the condition monitoring on the compressors to predict failures at earlier stages. Also, limited studies went through the modern maintenance engineering practices to come with high equipment effectiveness [1]. Study was a recent review of applied modern condition monitoring and best maintenance engineering practices in gas compression plants.

Failure modes and effects analysis (FMEA) determines the effect of each failure mode and its causes on the system or equipment based on the severity (S), occurrence (O) and detectability (D). The measurement of RPN is shown Eq. (1) in below: -

(1) RPN=Severity×Occurance×Detectability

where “S” mean severity, which is a non-dimensional number. Severity identifies the single failure mode which strongly affects the system performance. “O” means an occurrence which depends on the probability of occurrence of defect in the system during the exposure time. “D” means detection ways and the ability to identify the failure modes. RPN calculated on the basis of severity and occurrence rank only. Higher values of RPN mean that particular defects mainly affect the system performance [2], [3], [4].

A severity rank of failure mode depends on the degradation rate per year and safety issues. The severity number range is related to safety issues and highest degradation factor, whereas the used numbers depend on the performance degradation factor [5], [6], [7]. It is very difficult to find out the severity rank of a particular failure mode, as the degradation of a module is a cumulative sum of many factors.

Kiran [8] Research defined the risk priority number (RPN) is a function of the three parameters which are the severity, the probability of occurrence and the detection sense and its calculation. Moreover, they explained that the RPN may help in indicating the threshold values for determining the areas of greatest concentration which needs a knowledge of the system behaviour along with the determination of the modes of failure with higher RPNs priorities [9]. FMEA method is used to calculate in particular the related RPN for each failure mode and then proposed recommended actions to reduce its associated risk [10].

Sellappan et al. [11] Study developed an effective risk prioritization method to enhance the common FMEA process. Result focused to ensure having high quality and reliability of the products by re- design the FMEA. Their data were proposed a modified risk to deal with subjective and qualitative information in their proposed framework and their result demonstrated the potential of the modified prioritization of failure modes in a ranking scale by using software using risk priority code (RPC).

Murri et al. [12] Research elaborated on the basic structure of a system and particularly from those system elements for which accurate information about failure mode and its causes. By analysing the functional relationships among these elements, it identified the possibility of propagating each type of failure to predict its effects on the production performance of the entire system. Their study was an inductive method to analyse failure modes using down-top methodology.

The alleged reliability led the longest warranty period for Photovoltaic (PV) modules up to 25 years which became possible after understanding the failure mode and degradation analysis of PV module. Failure mode decreases the performance of the PV module throughout the long-term outdoor exposure [13]. The main objective of the study was to identify the failure mechanism of a solar PV modules and their impact on degradation in operating scenarios. Identified that, The RPN analysis was to identify the single failure mode which impact a particular performance in solar system.

Kim and Zuo [14] Study presented a general model to explain the functional relationship among the three factors of RPN and applied in model for demonstration and discussed the unique role of each factor for comparing the risk of different failure modes [15]. The extended the definition of RPN by multiplying it with a weight parameter which characterize the importance of the failure causes within the system. Finally, the effectiveness of the method was demonstrated with numerical examples.

Dhillon [16] RPN technique was also applied and used in the automotive industry to prioritize their failure modes. [17] A new methodology for Laboratory Assessment and Risk Analysis in research environment (LARA) and developed a new risk index called Laboratory Criticality Index (LCI) for risk ranking. LCI is conceived through two approaches which are the Risk Priority Number (RPN) and the Analytic Hierarchy Process (AHP) which provided the identification of critical areas and prioritization of safety actions. [18] A presented research aimed to propose a new method called Total Efficient Risk Priority Number (TERPN) to classify risks and to identify corrective actions in order to obtain the highest risk reduction with the lowest cost. They suggested a suitable model for ranking risks in a company to reach the maximum effectiveness of prevention and protection strategies. The TERPN method was an integration of used failure mode effect and criticality analysis (FMECA) with the risk assessment factors.

Moreover, RPN technique is also used to study cases in medical fields to prioritize healthcare system failures [19]. A study highlighted the patient's journey in surgery ward from holding area to the operating room. The highest priority failures determined for clinical effect, claim consequence, waste of time and financial loss). The risks priority criteria quantified by using RPN index and the ability of improved RPN scores were reassessed by root cause analysis.

## Methods of failures’ ranking

From the operational log sheet and register, it is observed several failures recorded which caused impact to the main processing plant. Appendix I is showing the 34 recorded failures for the past four years which is high for such processing gas plant need continuous compression.

There are several possible ways to sort and rank the operational failures list for a plant and machine. Each method can rank based on the required concept, thus there are two methods will be customized for to rank these 34 failures based on the most contributed failures into the total down time and based on the high risk failures.

### Total down time importance (TDTI)

TDTI is a new proposed method to obtain the overall contributor effects into components. TDTI gives the weight of each failure down time effect into the total occurred down time in the system.

This gives a better indication of the criticality and importance of the failure mode contribution and effect in the overall system. Therefore, the TDTI shows the failure mode contribution compared to the total down time. Table 1 presents the top down time contributors as TDTI which determined for a high pressure (HP) gas compressor package in oil and gas plant. The TDTI concept is to consolidate a huge failures datasheet into controllable dedicated groups. This is made by investigate the main root causes and consequent source of failure occurrence.The TDTI is applied and summarized the list of the 34 recorded failures as shown in Appendix I into 11 groups based on their contribution to the total down time of the HP package. The 11 groups are sorted based on the origin source and bases of the failures effects on the total down time.

**Table 1:** Total down time importance ranking.

ID	TDT Importance Hours	TDT Importance Accumulative	TDT Importance%	TDT Importance% Accumulative
Vibration	3360	3360	30.43%	30.43%
DNFT (Digital No Flow) device	2640	6000	23.91%	54.34%
Control Issue	1032	7032	9.35%	63.69%
Cylinder High Temperature	1008	8040	9.13%	72.82%
Blockage Drain	840	8880	7.61%	80.43%
Lube Oil	672	9552	6.09%	86.53%
Automation and Instrument	624	10,176	5.65%	92.17%
Motor	480	10,656	4.35%	96.52%
Isolation Valve	168	10,824	1.52%	98.04%
High Temperature	168	10,992	1.52%	99.57%
Belt Rupture	48	11,040	0.43%	100.00%
**Total**	**11,040**	**96,552**	100%	

### Risk priority number (RPN)

**Fig. 1:**
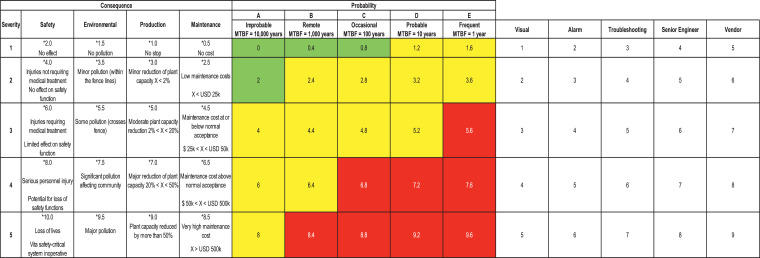
The organization risk matrix.

Continuous consideration of risk nowadays plays a core role since basic design, development and while operation. In all generality, the problem of risk arises wherever there is an existing potential source of hazard. The area of study organization has their own criticality matrix as shown in Fig. 1.

#### Detectability

Detectability means a change in behaviour of the asset prior to the failure. Temperature, speed, vibration, noise changes somehow warning the operator by an alarm.

Some failure modes are relatively easy to spot and others require diagnostic work to isolate them, troubleshooting. Therefore, as a part of modern reliability cantered maintenance (RCM) determination and calculating the failure mode effects and criticality assessment (FMECA), it is important to assess the risk priority number (RPN). In other words, a failure mode with a high RPN number should be given the highest priority in the analysis and corrective action.

#### Consequence severity

The consequence severity is an assessment of the significance of the failure mode's ‘Global’ effect on a system's operation with respect to production loss (downtime). Severity will be evaluated while taking mitigation factors into account. The severity levels are defined in 5 levels in the organization risk matrix as shown in figure [1] with consequence impacts on Safety, Environment, Production and Maintenance.

#### Detectability

Detectability means a change in behaviour of the asset prior to the failure. Temperature, speed, vibration, noise changes somehow warning the operator by an alarm.

Some failure modes are relatively easy to spot and others require diagnostic work to isolate them, troubleshooting.

The options of means of detections are as following:

(1) Through sense: mostly visually but also by ear, smell or touch
(2) Through alarm: audible alarms, warning lights
(3) Operator: the asset operator has the knowledge and skills to identify the failure mode root cause
(4) Discipline engineer/technician/ specialists, health, safety and environment/all persons with the required skills to identify the failure mode root cause
(5) External experts: manufacturer/specialist brought in to discover the failure root cause

Note that the scores increase with the requirement for more experts’ mobilisation in the action team formed. Therefore, an Equipment Vendor brought in to the Root Cause Analysis will have the highest Risk Criticality Scores as shown in figure [1].

To demonstrate more, an example for an emergency shut down (ESD) Loop that initiating transmitter logic solver shutdown valve at the inlet as failure mode “Operates without Demand”. The determination basis as explanation is as in below:

(1) If Severity is 1 for Safety, 2 under Environmental, 3 under Production and 2 for Maintenance then the values to be used are 2, 3.5, 5 and 2.5 respectively. Now the maximum value will be considered which will be 5.
(2) For probability, the number shall be taken based on the Risk Ranking, example if B3, as likelihood is B and the Severity worst case is 3 (as given above for Production). So, B3 value to be considered is 4.4.
(3) For the Detectability, if the worst Severity number as 3 in our example, the corresponding maximum value is picked based on all the available Detectability in place (we have Visual as 3, Alarm as 4 and Troubleshooting as 5) so, Corresponding Detectability scores as also will be the maximum for the used calculation as 5. Hence the

RPN=5 × 4.4 × 5 = 110

Following the above mentioned consequence, all RPN is calculated and listed as shown in Appendix II.

## Result and discussion

**Fig. 2:**
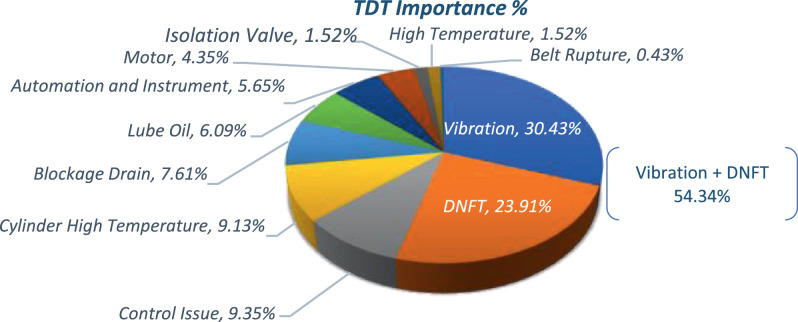
TDT importance grouping effects.

Based on the overall RCM exercise, equipment items and failure modes that have been identified as critical, based on Risk Priority Numbering (RPN). RPN may not play an important role in the choice of an action against failure modes, but will help in indicating the threshold values for determining the areas of greatest concentration. In other words, a failure mode with a high RPN number should be given the highest priority in the analysis and corrective action. Fig. 2 shows the results obtained while using the TDT importance and reduce the long data of failures into groups (from 34 failures into 11 groups).

So, reference to the main out comes of using TDTI and RPN ranking, here in below the major concerns and findings cause the highest and risk down time to the compression unit: -

Vibrational issues root-causes are still unknown. This led to the change several items and spare parts like piston rods, collars, pipes and crank shaft after short term. Potential causes could be due to skid, structure and piping layout. By calculating the TDTI, it shows that above 54% of downtime causes are due to the vibrational impacts and the DNFT trips which means these two concerns are the main issues to focus on rather than others.

Frequent Compressor trips due to Digital No Flow Timer (DNFT) trip signal. The major causes have been weak lubrication feed and minor electrical issues for which manufacturer review to resolve the issue. Also, the screw from demister packing falls frequently to the bottom which cause blockage to the drainage of the condensate gas need engineering review for the method of the tie in and connection.

The Scrubber design and re-size calculations need to be revising to enable handling the incoming gas amount.

Due to gas quality, there is an accumulation of moisture and condensate in the compressor. This leads to further studies in the Gas Compressor inlet.

## Conclusion

There are many ways to rank and sort a list of failures based on the required aims and needs of enhancement. Most common needs are either related to risk effects on the unit or related to failures which cause high downtime in order to resolve.

The TDT importance has the philosophy to summarize a long list of operation failures by grouping the failures based on the contribution of the down time into the equipment. By using the TDT importance in the study, these 34 failures have been grouped into 11 sections which lead to where to focus like it is found the vibration and DNFT are causing 54.6% from the overall downtime. So these both sectors (vibration and DNFT) need further analysis and more improvement studies as well the scrubber resizing calculation in also required.

Also, list by calculating the risk priority number (RPN) showed the failures ranking related to each associated risk and failures impacts. The ranking need close and frequent monitoring and measuring as it will continuous change due to the associated severity, impact and changeable operating conditions.

These two methods can be customized and used to analyse further ranking of the failures and priorities for any records in industrial premises or other manufacturing entities with the same concept, so it is recommended to apply several customizations in different fields like manufacturing, medial sectors, agricultures, etc. to ensure getting the analysis of down time grouping and raking compared to work business natures and site environment..

## Declaration of Competing Interest

This is to declare that, this study is for scientific outcomes in order to study building an integrated maintenance frame work for post graduate studies. This study was not funded by any entity or organization and it is mainly a core outcome from the research and will not cause any conflict of interests by any means.

## Figures and Tables

**Table:** 

Fault	Cause	Down time (days)
Lube oil temperature high	Thermostat valve had stuck	20
Pre-lube oil pump overload relay trip	The pre lube oil pump overload relay faulted	7
Air Cooler motor vibration high	vibration switch set point not suitable	1
2nd stage discharge scrubber cannot drain	condensate outlet pipeline blocked	35
Local Control Panel communication with DCS failure for short time.	The fault was from the control switch.	8
Nitrogen regulator pressure gauge not reading	Found regulator isolation valve is block	7
Control room found gas flow rate is 0, but DCS display HP gas compressor status is normal,	Local control panel fault, fuse of the power supply of the module blown.	1
Cylinders temperature found running with high temperature	Unloader valves and discharge valves were leakage	2
air cooler fan motor Over load relay trip frequently	Panel very hot	12
Compressor tripped due to high temperature	valve pin for divider block was stuck	7
Compressor failed to start up	Fault DNFT signal	1
1st stage suction scrubber Temperature reading Zero	Analogy card problem	12
The signal was not showing in the compressor HMI.	Two Channels in the control card were showing fault indication.	1
Compressor tripped due to DNFT	Fault DNFT signal	26
Cooling fan motor No.3 running with abnormal noise	NDE side running with high noise	7
Cylinder No.4 running with high temperature	Two discharge valve and one unloader were leakage	5
PLC program crash notification.	Can cause by many reason like example power trip	2
Temperature transmitter was showing no reading in the Gas Compressor HMI.	The control card was showing fault indication.	1
Air cooling fan No. 2 & 3 stopped	belts ruptured	1
Cylinder No.2 running with high temperature	Discharge valves No. 1 found leakage	13
Cylinders No.3&4 high temperature.	Discharge valve No. 1,2 leakage for cylinder No. discharge valve No.1&2 were leaking	7
Compressor tripped due to DNFT	Fault DNFT signal	4
Suction & Discharge Scrubbers TITs not giving correct reading	Transmitter length too short for sensing	9
Compressor tripped due to DNFT	Fault DNFT signal	8
The time in the gas compressor HMI not matching with DCS System.	Time delay happening when the compressor LCP shutdown.	18
Air cooling fan #2 stopped	belts raptured	1
Cylinder No. 4 due running with high temperature	Two discharge valve and one unloader were leakage	1
A Lube oil cooler fan motor MCB tripped	Main Control panel tripped	1
Compressor tripped due to alarm	High High alarm signal	140
The signal for cylinder No 2 showing wrong reading in the HMI.	Faulty signal	8
Compressor tripped due to alarm in suction scrubber	suction scrubber Level Transmitter high high Alarm	9
Cylinder No. 3&4 running with high temperature	Two discharge valve for cyl#4 and one discharge valve for cy#3 were leakage	1
1st cylinder No.1 and 2nd cylinder No.2&4 were running with high temperature.	1st one discharge valve was leakage and 2nd stage two discharge and suction valve were leakage	13
Compressor tripped due to DNFT	weak lubrication feed, lead to DNFT trip signal	71

**Table:** 

RPN	Equipment Item and Failure mode
828	Gas Compressor Vibration
756	Gas Compressor Overheat
588	Emergency shut down (ESD) loop Fail to close on demand
588	Emergency shut down (ESD) loop Fail to close on demand
529.2	Operates without demand
528	Emergency shut down (ESD) loop Fail External leakage
528	Pressure control valve & Pressure Control Loop External leakage
528	Emergency shut down (ESD) loop External leakage at inlet of 1st stage Suction Scrubber
528	Level control valve & Level Control Loop External leakage
528	Emergency shut down (ESD) loop External leakage at outlet of 2nd stage Suction Scrubber
528	Level control valve & Level Control Loop External leakage
528	Emergency shut down (ESD) loop External leakage at outlet of 2nd stage Discharge Scrubber
528	Level control valve & Level Control Loop External leakage
528	Emergency shut down (ESD) loop External leakage at outlet of Gas Compressor
504	Suction Scrubber (1st Stage)External leakage
504	Gas Compressor Partial or total blockage of compressor suction
504	Suction Scrubber (2nd Stage) External leakage
504	Discharge Scrubber (2nd Stage) External leakage
425.6	Suction Scrubber (1st Stage) Plugged/choked
425.6	Compressor Breakdown
409.6	After Cooler Abnormal instrument reading
374.4	Suction Scrubber (2nd Stage) Plugged/ choked
333.2	Gas Compressor Fail to start on demand
313.6	Gas Compressor Motor Fail to start on demand
256	Gas Compressor Motor Overheating
256	Gas Compressor Motor Vibration
256	(Stage 1) After Cooler Insufficient heat transfer
182	Discharge Scrubber (2nd Stage)Plugged/choked
156	Gas Compressor Low Output
110	Emergency shut down (ESD) Operates without demand at Inlet of Gas Compressor
110	Emergency shut down (ESD) Operates without demand at inlet of 1st stage Suction Scrubber
110	Emergency shut down (ESD) Fail to close on demand at inlet of 1st stage Suction Scrubber
110	Emergency shut down (ESD) Operates without demand at outlet of 2nd stage Discharge Scrubber
110	Emergency shut down (ESD) Failure to close on demand at outlet of 2nd stage Discharge Scrubber
104	Gas Compressor Motor Low Output
96	Gas Compressor Motor Breakdown
88	Pressure control valve & Pressure Control Loop for minimum circulation Fail to open on demand
88	Pressure control valve & Pressure Control Loop for minimum circulation Fail to close on demand
88	Level control valve & Level Control Loop Fail to open on demand
88	Level control valve & Level Control Loop Fail to close on demand
88	Emergency shut down (ESD) operates without demand at outlet of 2nd stage Suction Scrubber
88	Emergency shut down (ESD) Fail to close on demand at outlet of 2nd stage Suction Scrubber
88	Level control valve & Level Control Loop Fail to open on demand
88	Level control valve & Level Control Loop Fail to close on demand
88	Failure to open on demand
88	Failure to close on demand
56	Abnormal instrument reading
48	Abnormal instrument reading
42	Abnormal instrument reading
36	Abnormal instrument reading
36	Abnormal instrument reading
28.8	Abnormal instrument reading
21.6	(Stage 2) After Cooler Insufficient heat transfer
